# ENaC in Salt-Sensitive Hypertension: Kidney and Beyond

**DOI:** 10.1007/s11906-020-01067-9

**Published:** 2020-08-27

**Authors:** Ashley L. Pitzer, Justin P. Van Beusecum, Thomas R. Kleyman, Annet Kirabo

**Affiliations:** 1 Division of Clinical Pharmacology, Department of Medicine, Vanderbilt University Medical Center, 2215 Garland Avenue, P415C Medical Research Building IV, Nashville, TN 37232 USA; 2 Department of Molecular Physiology and Biophysics, Vanderbilt University, Nashville, TN USA; 3 Departments of Medicine, Cell Biology, Pharmacology, and Chemical Biology, University of Pittsburgh, Pittsburgh, PA USA

**Keywords:** ENaC, Hypertension, Inflammation, Sodium

## Abstract

**Purpose of Review:**

The main goal of this article is to discuss the role of the epithelial sodium channel (ENaC) in extracellular fluid and blood pressure regulation.

**Recent Findings:**

Besides its role in sodium handling in the kidney, recent studies have found that ENaC expressed in other cells including immune cells can influence blood pressure via extra-renal mechanisms. Dendritic cells (DCs) are activated and contribute to salt-sensitive hypertension in an ENaC-dependent manner. We discuss recent studies on how ENaC is regulated in both the kidney and other sites including the vascular smooth muscles, endothelial cells, and immune cells. We also discuss how this extra-renal ENaC can play a role in salt-sensitive hypertension and its promise as a novel therapeutic target.

**Summary:**

The role of ENaC in blood pressure regulation in the kidney has been well studied. Recent human gene sequencing efforts have identified thousands of variants among the genes encoding ENaC, and research efforts to determine if these variants and their expression in extra-renal tissue play a role in hypertension will advance our understanding of the pathogenesis of ENaC-mediated cardiovascular disease and lead to novel therapeutic targets.

## Introduction

The balance between salt and body fluid volume is necessary for regulating blood pressure. High blood pressure is the leading cause of morbidity and mortality due to cardiovascular-related diseases, such as stroke, heart failure, myocardial infarction, and chronic kidney disease [1]. Reducing dietary intake of sodium (Na^+^) decreases both hypertension and rate of morbidity and mortality associated with cardiovascular events [2]. A meta-analysis investigating the long-term effects of dietary salt intake on blood pressure showed that a reduction of salt for 4 weeks or more results in a significant reduction in blood pressure regardless of sex or ethnic group. A major problem with excess salt consumption is that approximately 25% in the general population and nearly half of the hypertensive population are salt-sensitive [3]. Salt-sensitivity is defined by the hyperresponsive increase and decrease in blood pressure to salt loading and salt depletion, and is an independent predictor of death and cardiovascular events [4, 5]. In contrast to salt-resistant individuals, those who are salt-sensitive experience abnormal changes in blood pressure in response to even minor changes in plasma salt levels [6].

Recently, a paradigm-shift in the understanding of Na^+^ handling has elucidated a role of extra-renal interstitial space [7, 8]. Studies using ^23^Na MRI and mathematical modeling demonstrated high Na^+^ content in the skin and muscle junctional zones positively correlates with blood pressure in humans [9–11]. These observations regarding tissue Na^+^ have relevance to immune cell activation which contributes to hypertension since monocytes can enter and re-emerge from tissues with minimal or no differentiation [12]. There is strong evidence that monocytes contribute to both blood pressure elevation and end-organ damage associated with hypertension. Deletion of monocytes markedly reduces experimental hypertension [13]. Cells derived from monocytes, including macrophages and DCs have also been implicated in hypertension [14, 15, 16••]. Despite the extensive studies, the mechanisms mediating tissue sodium-induced activation of immune cells and hypertension are still not well understood.

**Fig. 1:**
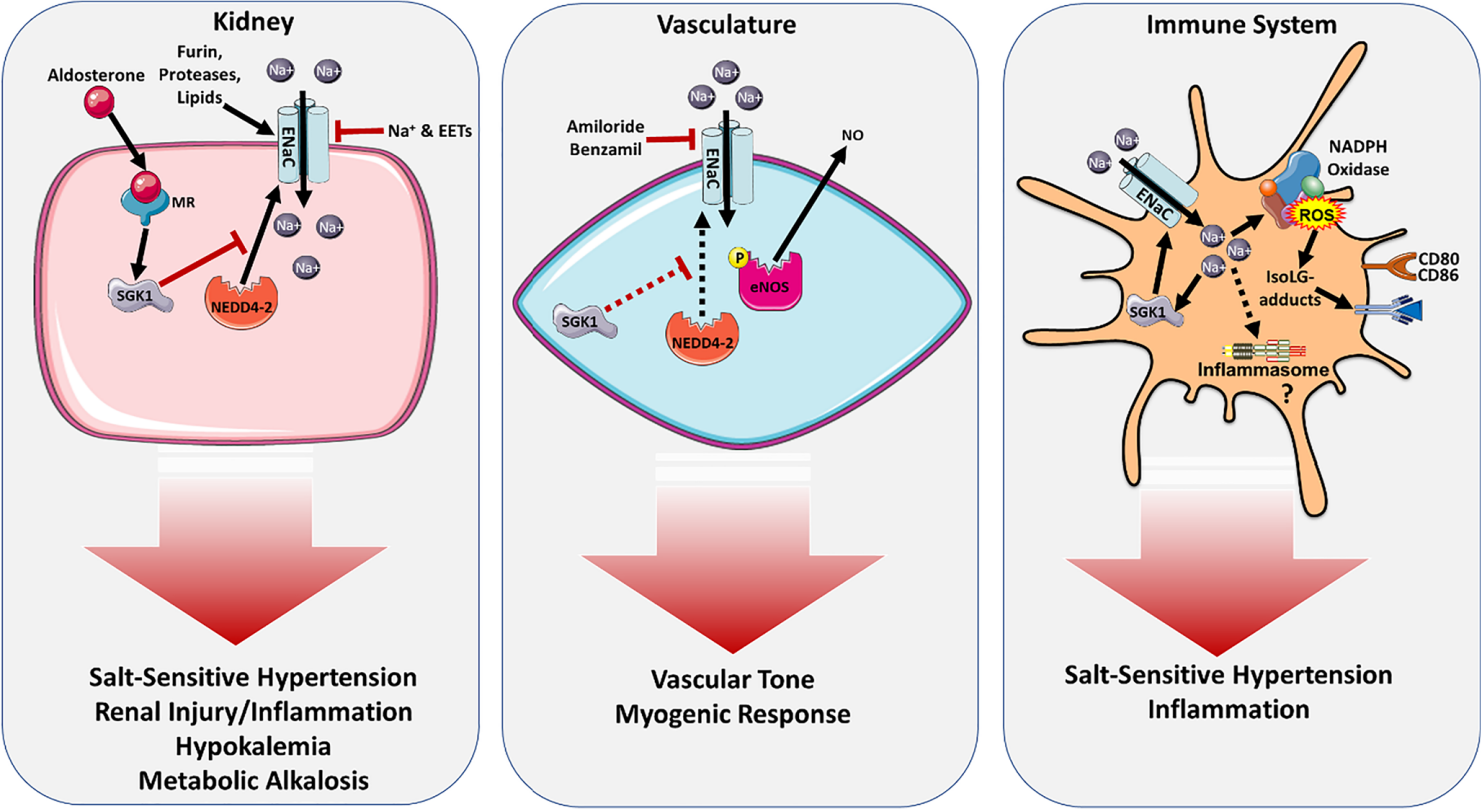
ENaC regulation in the kidney, vasculature, and immune system. ENaC expression and activation in renal distal tubule epithelial cells is regulated by hormonal factors, proteases, lipids, and select ions promoting hypertension, renal injury and inflammation, hypokalemia, and metabolic alkalosis. In vasculature, inhibition of ENaC leads to increased nitric oxide production mediating vascular tone and myogenic response. Activation of ENaC in innate immune cells stimulates ROS production, pro-inflammatory cytokine secretion, and antigen presentation resulting in inflammation and salt-sensitive hypertension

The epithelial Na^+^ channel (ENaC) plays a critical role in body fluid volume and Na^+^ homeostasis which underly the pathogenesis of salt-sensitive hypertension [17, 18]. This channel has been extensively studied in the kidney where it plays a role in controlling Na^+^ and K^+^ handling (Fig. 1). However, it is expressed in other tissues such as the endothelium, vascular smooth muscle, tongue, colon, and immune cells and has been found to influence blood pressure via extra-renal mechanisms. Here, we review the mechanisms of the ENaC-mediated Na^+^ balance and its relationship to salt-sensitive hypertension and inflammation. Understanding the relationship between salt and the predisposition for high blood pressure could provide valuable insight in drug development for the prevention and treatment of hypertension.

### ENaC: Kidney and Beyond

ENaC belongs to the ENaC/degenerin family of ion channels which are sensitive to extracellular factors. ENaC is typically a heterotrimer consisting of three homologous subunits: α, β, and γ [19–21]. A fourth subunit, δ, is functionally similar to the α-subunit and is found in various epithelial and non-epithelial tissues of humans such as in the pancreas, lung, and brain [22–25]. Unlike other subunits, the α-subunit is able to form a homo-trimeric channel that is able to conduct Na^+^. Co-expression of all three subunits (α, β, and γ; or δ, β, and γ) is required to attain full channel activity [20]. αβγ and δβγ have different functional properties. For example, αβγ channels are inhibited by extracellular Na^+^, and full activation requires furin-mediated proteolytic processing in the trans-Golgi network and at the cell surface by specific proteases, referred to as channel activating proteases (CAPs) [26–28]. In contrast, human δβγ channels are largely insensitive to extracellular Na^+^ and are not activated by proteases [22, 29]**.**

#### Mechanisms of ENaC Regulation

##### Na^+^ Self-Inhibition

Elevated extracellular Na^+^ inhibits ENaC through two different mechanisms. First, extracellular Na^+^ binds ENaC at a defined site in the α-subunit and drives an allosteric change reducing the ENaC open probability, which is referred to as Na^+^ self-inhibition [30, 31]. This self-inhibition is rapid, low-affinity, and cation-selective. Not only can Na^+^ inhibit ENaC activity, but Li^+^ is also able to have an inhibitory effect while K^+^ only has a minimal inhibitory effect [26, 32, 33]. Second, increased intracellular Na^+^ slowly inhibits the channel over time [34, 35]. In cultured cells, increases in intracellular Na^+^ renders channels insensitive to cleavage and activation by proteases [36, 37]. ENaC regulation by Na+ self-inhibition (NaSI) enables the distal nephron to control Na^+^ reabsorption based on fluctuating urinary Na^+^ concentrations. Numerous studies have found that many of the human ENaC nonsynonymous single nucleotide variants affect channel function by altering the NaSI response [38–42].

##### ENaC Regulation by Post-Translational Proteolytic Cleavage

Another mechanism by which ENaC is regulated is through post-translational proteolytic cleavage at defined sites in their extracellular domains with release of imbedded inhibitory tracts leading to activation of the channel through an increase in channel open probability [28, 39, 43]. The ENaC subunits αβγ are assembled and processed in Golgi and post-Golgi compartments and are cleaved by furin [28, 43, 44]. The α-subunit is cleaved twice by furin, releasing an inhibitory tract and transitioning channel from a low to an intermediate activity state. Furin cleaves the γ-subunit once. Its cleavage by other proteases including prostasin, matriptase, cathepsin B, elastase, kallikrein, urokinase, and plasmin at sites distal to its inhibitory tract releases the tract and transition channels to a high activity state [43, 45–53]. Other key regulatory factors interface with ENaC subunit proteolysis to determine channel open probability [27•].

##### ENaC Regulation by Lipids

Lipid signaling molecules including phosphatidylinositol 4,5-bisphosphate and phosphatidylinositol (3,4,5)-trisphosphate have been found to enhance ENaC open probability by binding to cationic sequences within ENaC subunits [54–56]. In contrast, the CYP-epoxygenase metabolite 11,12-epoxyeicosatrienoic acid (EET) inhibits ENaC by reducing its channel open probability [57, 58]. Post-translational modification of specific cytoplasmic Cys residues on the β- and γ-subunits by palmitoylation is another mechanism by which ENaC is activated by lipids [59, 60].

##### ENaC Regulation by Aldosterone

Aldosterone regulates ENaC activity through a variety of mechanisms, including an increase in expression and, in concert with other hormones, activation of a serum-and-glucocorticoid-induced kinase (SGK1) [61, 62]. Aldosterone is secreted from the adrenal gland in response to salt loss and volume depletion, and in response to an elevated serum [K^+^] [63, 64]. The binding of aldosterone to the mineralocorticoid receptor in renal epithelial cells activates SGK1, which in turn phosphorylates NEDD4-2, a ubiquitin ligase that interacts with ENaC at the cell surface through carboxyl-terminal Pro-Tyr motifs on channel subunits. Ubiquitination of ENaC subunits targets the channel for internalization and degradation. Phosphorylation of NEDD4-2 recruits a 14-3-3 protein and prevents the interaction of NEDD4-2 with ENaC, contributing to the accumulation and enhanced expression of ENaC at the plasma membrane, as well as an increase in channel open [65–67].

### ENaC Function in the Kidney

Classically, ENaC has been demonstrated to be involved in reabsorption of filtered Na^+^ in the distal nephron, including the aldosterone-sensitive distal nephron (ADSN) and the collecting duct [68–73]. In the kidney, ENaC acts as the final and rate-limiting step in determining transepithelial Na^+^ reabsorption, net total body Na^+^ content, fluid volume, and blood pressure in the collecting duct of the nephron. ENaC is primarily expressed in the apical membrane of late distal tubule epithelial cells, which is also known as the ASDN. In addition to its role in Na^+^ reabsorption, ENaC also plays a critical role in the secretion of K^+^ in the ASDN [31, 74, 75]. The discovery of mutations in the human ENaC channel confirmed the role of ENaC in regulating blood pressure homeostasis. These ENaC mutations lead to Mendelian forms of hypertension or hypotension, Liddle syndrome, and pseudohypoaldosteronism type 1 (PHA1), respectively. Liddle’s syndrome occurs through a gain-of-function mutation in the cytoplasmic C-terminus of either the β- or γ-subunit of ENaC resulting in an autosomal-dominant form of salt-sensitive hypertension, hypokalemia, and metabolic alkalosis through constitutively active ENaC [76]. In this setting, hypokalemia is predicted to activate the Na/Cl co-transporter, which also contributes to the hypertension in Liddle syndrome [77]. Conversely, inherited loss-of-function mutations in ENaC result in PHA1 and present physiologically as severe hypotension, renal salt wasting, metabolic acidosis, and hyperkalemia [78–81]. A recent study has shown that treatment of hyperaldosteronism with low-dose amiloride, a pharmacological inhibitor of ENaC activity, normalized previously elevated blood pressure within 1–4 weeks of starting the amiloride treatment and was maintained for 14–28 years [82]. In cases of uncontrolled hypertension, it has been demonstrated that mineralocorticoid receptor blockade with spironolactone was sufficient in reducing systolic blood pressure regardless of their levels of aldosterone. In many cases where ENaC activity is constitutively active, the Na^+^ channel can be inhibited by the K^+^-sparing diuretic, amiloride [83–85]. For instance, a population of African-Americans was resistant to the blood pressure–lowering effects of spironolactone but had a significant reduction when treated with amiloride [86]. This evidence suggests a therapeutic role for targeting hyperactive ENaC activity in hypertensive patients.

### ENaC Beyond the Kidney

#### Vascular Smooth Muscle Cells

In addition to the ASDN and collecting duct, certain subunits of ENaC have been shown to mediate vascular tone through their expression in vascular smooth muscle cells. It has been demonstrated that all ENaC subunits (α, β, and γ) are expressed in mesenteric, cerebral, and renal arteries [23, 87–89]. Perez et al. showed that all ENaC subunits are expressed in rat mesenteric resistance arteries and elegantly showed that inhibition of ENaC using either benzamil or amiloride increases the ratio of phosphorylated epithelial nitric oxide synthase (p-eNOS)/total eNOS through a phosphoinositide 3-kinase (P13K)/Akt-dependent mechanism [87]. Moreover, within the cerebral vasculature, Drummond et al. demonstrated by mRNA and protein expression that both β- and γ-subunits of ENaC are expressed in cerebral resistance arteries and that inhibition of ENaC with amiloride or benzamil prevented pressure-induced vasoconstriction [23]. Within the kidney, Guan et al. demonstrated that vascular smooth muscle cells from afferent arterioles express α, β, and γ ENaC subunits. They went on to demonstrate that micromolar doses of amiloride or benzamil, which do not affect l-type calcium channels, inhibited afferent arteriole myogenic response [88]. To investigate which subunit of ENaC contributes to the myogenic response in resistance vessels, Ge et al. used a mouse model of reduced βENaC expression (βENaC m/m). They showed that reduction of βENaC led to the impairment of whole kidney renal autoregulatory capability [89]. Taken together, these studies suggest that ENaC plays a fundamental physiological role in vascular smooth muscle cell function and regulation of blood flow through resistance vessels by modulating the myogenic response.

#### Vascular Endothelium

All 4 subunits of ENaC (α, β, δ, and γ) have been demonstrated to be expressed in the vascular endothelium by mRNA transcription and/or protein expression by immunofluorescence [90–97]**.** Interestingly, there is evidence that endothelial ENaC can be regulated much likely in the kidney. For example, Oberleithner and colleagues showed that nanomolar concentrations of aldosterone increases the expression of ENaC subunits by approximately 36% and total cellular ENaC by 91% in human umbilical vein cells (HUVECs) [94]. Moreover, co-administration of amiloride and aldosterone leads to an 84% reduction in total ENaC in HUVECs, suggesting a regulation mechanism similar to the kidney. However, the way that endothelial ENaC handles extracellular Na^+^ differs from the renal epithelium. For instance, increases in extracellular Na^+^ content leads to downregulation of ENaC within the renal epithelium. In contrast, in endothelial cells, aldosterone plus high extracellular Na^+^ (beyond 140 mM) increases ENaC protein expression within minutes [91]. The precise mechanism by which this occurs is currently unknown, although it is hypothesized that in endothelial cells, aldosterone and high extracellular Na^+^ environment activates SGK1 which in turn phosphorylates NEDD4-2 which renders it inactive. Thus, ENaC plays a critical role in both intra- and extra-renal vascular function.

### ENaC Activity and Regulation in Immune Cells

Activation of the adaptive immune system in hypertension may occur through the loss of self-tolerance, suggesting it may be an autoimmune disease. Antigen-presenting cells (APCs), including macrophages, DCs, and B cells, are critical initiators of the immune response. Of these APCs, DCs are the most proficient classical antigen presenters and play an important role in the discrimination between self and non-self-antigens. In 1973, Ralph Steinman first discovered and described DCs, and since their discovery, they have been extensively studied and well characterized as potent stimulators of T cell activation [98]. APCs present antigens that are then recognized by T cell receptors, stimulating T cell proliferation and activation. Various hypertensive stimuli including angiotensin II (Ang II) and norepinephrine and dietary salt stimulate the infiltration of monocytes, macrophages, DCs, and T lymphocytes into the vasculature and kidney to promote Na^+^ retention, blood pressure elevation, vasoconstriction, and end-organ damage [14, 99–103]. Until now, salt sensitivity studies have focused on the roles of the vasculature, kidney, and sympathetic activity; however, the contribution of immune cells remains largely unknown.

Several studies have identified multiple neoantigens, or modified endogenous molecules no longer identified as “self,” for their potential to trigger the adaptive immune system. It is thought that these molecules are modified by post-translational modification, adduct formation, oxidation, and nitrosylation. One particular molecule that has been intensely studied for over 40 years for its potential role in hypertension and transport and delivery of antigenic peptides is heat shock protein 70 (HSP70) [104]. Only recently has HSP70 been suggested to induce an autoimmune reaction leading to T cell activation and polarization of CD4^+^ into regulatory T cells in salt-sensitive hypertension [105, 106]. Additionally, the Toll-like receptor 9 (TLR9) expressed in the endoplasmic reticulum of immune cells recognize mitochondrial DNA-derived cell-free unmethylated CpG dinucleotides, which are upregulated in patients with essential hypertension [107]. Internalization of these CpG motifs activates the TLR9 signaling cascade through pro-inflammatory transcriptional factors NF-κB and AP-1 [108]. The ability for TLR9 to discriminate between methylated and unmethylated DNA is critical in preventing an autoimmune reaction. Moreover, studies in our laboratory have found that a new neoantigen in APCs contributes to the development of hypertension, its associated inflammation, and end-organ damage [16••]. We established a critical role of reactive oxygen species (ROS) and nicotinamide adenine dinucleotide phosphate (NADPH) oxidase in activating DCs in hypertension and in the modulation of gene expression and immunogenicity. In response to a hypertensive stimuli including Ang II, DOCA-salt, or N-nitro-l-arginine methyl ester (l-NAME/high-salt feeding), gamma-ketoaldehydes known as isolevuglandins isoLGs are formed in DCs [16••]. Importantly, scavenging of IsoLG-adducts attenuates blood pressure, inflammation, and vascular stiffness [109].

Recently, we investigated the signaling mechanisms of Na^+^-dependent ENaC activation in DCs [14]. We demonstrated that an increase in extracellular Na^+^ concentrations leads to an ENaC-dependent activation of the NADPH-oxidase and subsequent superoxide production leading to formation of the highly immunoreactive IsoLGs. Moreover, in monocyte-derived DCs, the production of IsoLG-adducted proteins can lead to loss of immune tolerance in DOCA-salt hypertension [14, 16]. This ENaC-dependent increased formation of IsoLG-adducted proteins in DCs after exposure to high salt correlates with an increase in surface expression of B7 ligands CD80 and CD86 indicating DC maturation and is essential for the pathogenesis of hypertension [16••]. These studies suggest a potential relationship between innate immunity, ENaC, and hypertension.

One important intracellular enzyme induced by Na^+^ is SGK-1. The role of SGK-1 in modulating blood pressure has predominantly been studied in the distal convoluted tubule where it regulates ENaC expression. Recent work in our lab has demonstrated that in APCs, the salt-sensing kinase SGK-1 mediates salt-sensitive hypertension by regulating increased expression of ENaC α- and γ-subunits, which leads to IsoLG-adduct formation, interleukin-1β (IL-1β) production, and T cell activation [110•]. Studies by Kleinewiefeld et al. and Wu et al. showed that when exposed to elevated Na^+^ concentration, there is a marked induction of Th17 polarization in naïve T cells [111, 112]. Inhibiting SGK-1 prevented activation of Forkhead box protein O1 and subsequent differentiation to the Th17 phenotype. In addition, SGK-1 signaling inhibits FOXP3^+^ regulatory T cells [113, 114], and both Th17 and regulatory T cells play a role in autoimmune tolerance and the genesis of hypertension [115, 116].

### IL-1β and ENaC: New Mechanism for Salt-Sensitive Hypertension?

In recent years, significant research progress has been made to better understand the relationship between inflammation and the pathogenesis of hypertension [117, 118]. Both animal and human studies suggest that cytokines such as IL-1β induce a pro-inflammatory state potentiating blood pressure elevation through the alteration of renal, endothelial, and immune responses [99]. In mice, targeting IL-1β activity has been shown to decrease blood pressure through pharmacological inhibition, IL-1β targeted antibody treatment, and genetic deletion [119–121]. In a recent article by Rothman et al., secondary analysis of the CANTOS trial suggested that while IL-1β inhibition with canakinumab reduced cardiovascular event rates, this benefit may not be related to incident hypertension and raises the question of the importance of inflammation in hypertension and development of cardiovascular disease [122•]. Moreover, there is a connection between high-salt environments and inflammation. Prior work by Shapiro and Dinarello showed that high salt concentrations drive peripheral blood mononuclear cells to produce the pro-inflammatory cytokine IL-1β [123]. Additionally, high salt increased ENaC-dependent production of IL-1β in DCs to mediate salt-sensitive hypertension by priming and polarizing T cells to produce interleukin 17-A (IL-17A) [14, 101, 124].

Although it is known that there are increased levels of circulating IL-1β in hypertension, only recently has the inflammasome activation been suggested to play a role in its production. Consisting of the sensing domain NOD-like receptor family, pyrin domain containing (NLRP3) and adaptor protein apoptosis-associated speck-like protein containing a carboxy-terminal caspase recruitment domain (ASC), the stimulated complex forms to recruit and proteolytically cleave pro-caspase-1 into the bioactive caspase-1. Caspase-1 activation results in the subsequent maturation and secretion of IL-1β [125, 126]. Hypertensive stimuli, including elevated Na^+^ and Ang II, are linked to ROS production which has been extensively studied for its role in inflammation, and recent evidence has shown that multiple sources of intracellular ROS are responsible for the activation of the NLRP3 inflammasome [127]. In a recent study by Krishnan et al., apoptosis-ASC^−/−^ mice were protected from DOCA-salt-induced elevated blood as well as renal inflammation and fibrosis [128]. Additionally, they demonstrated that pharmacological inhibition of the NLRP3 inflammasome abolishes DOCA-salt hypertension. In humans, elevated circulating levels of IL-1β and increased inflammasome gene expression have been correlated with age-related hypertension [129–131]. Moreover, mutations in the non-coding region of NLRP3 gene in humans were associated with susceptibility to developing hypertension [132]. Recently, ENaC-mediated Na^+^ influx was responsible for NLRP3 inflammasome activation in PBMCs of cystic fibrosis patients [133]. This suggests a possible link between increased Na^+^ content and IL-1β production in the pathogenesis of salt-sensitive hypertension. Current studies examining the inflammasome, its components, relationship to ENaC activity, and downstream effector cytokines provide promising insight into the role of salt and inflammation in the development of hypertension.

## Conclusion

In an effort to determine therapeutic targets for salt-induced hypertension, human gene sequencing efforts have identified several ENaC gain- and loss-of-function mutations that have been described in Mendelian disorders characterized by either hypertension or hypotension [134–144]. It is not known if individuals with gain-of-function ENaC variants have increased risk for salt-sensitive hypertension. Inhibition of ENaC using inhibitors such as amiloride is not a routinely used approach for treatment of hypertension given their low efficacy when compared other diuretics. However, a meta-analysis by Hebert et al. found that treatment of elderly hypertensive patients with ENaC inhibitors combined with a thiazide diuretic reduces coronary mortality and sudden cardiac death [145]. To date, most of the studies on ENaC have focused on its role in regulating renal Na^+^ and K^+^ handling. The recent seminal discoveries of the existence and functioning of extra-renal ENaC including immune cells may illuminate additional therapeutic targets for ENaC in salt-induced cardiovascular disease.
